# To what extent does the Tunisian law protect women against violence ?

**DOI:** 10.1192/j.eurpsy.2023.2399

**Published:** 2023-07-19

**Authors:** E. Mhiri, M. Maalej, I. Gassara, R. Feki, N. Smaoui, L. Zouari, J. Ben Thabet, S. Omri, N. Charfi, M. Maalej

**Affiliations:** 1 Psychiatry « C » department, CHU Hédi Chaker, Sfax, Tunisia

## Abstract

**Introduction:**

Tunisia, a pioneer in the Arab world in terms of promoting the status of women, has adopted a strategy to combat violence against women and now has a legal arsenal to protect women’s rights.

**Objectives:**

To study the contribution of the Tunisian jurisdiction in terms of protection of women victims of violence.

**Methods:**

A review of the different legal texts, using the key words: “Women”, “Violence”, “Jurisdiction” and “Protection”.

**Results:**

*The National Survey on Violence against Women in Tunisia is a long process that involved the efforts of several stakeholders.

*In 2011, Tunisia was the first country in the region to have ratified and lifted all specific reservations to CEDAW (Committee on the Elimination of Discrimination Against Women). Violence against women is considered a threat to peace and security.

*Secondly, the adoption of Article 46 of the new Constitution of 2014 obligated the State to combat violence against women and make it its priority.

*The adoption of Organic Law 58/17 of 11 August 2017 finally gave the status of victim to the abused Woman. This law came into force in 2018.

*A number of mechanisms have been put in place, including the establishment of the National Observatory to Combat Violence against Women.

*Most magistrates still resist the application of Law 58/17, which contributes to the resurgence of violence against women, especially during periods of slackening of the justice system, such as the COVID lockdown period.

**Conclusions:**

Despite the revolutionary legal arsenal acquired for the protection of women in Tunisia, there is still a gap between legislation and practice, and the rate of violence against women continues to increase. Nationwide awareness-raising campaigns aiming to spread awareness among women of their rights are necessary.

**Disclosure of Interest:**

None Declared

